# Development of Poly Lactic/Glycolic Acid (PLGA) Microspheres for Controlled Release of Rho-Associated Kinase Inhibitor

**DOI:** 10.1155/2017/1598218

**Published:** 2017-07-27

**Authors:** Sho Koda, Naoki Okumura, Junji Kitano, Noriko Koizumi, Yasuhiko Tabata

**Affiliations:** ^1^Department of Biomedical Engineering, Faculty of Life and Medical Sciences, Doshisha University, Kyotanabe 610-0321, Japan; ^2^Laboratory of Biomaterials, Department of Regeneration Science and Engineering, Institute for Frontier Life and Medical Sciences, Kyoto University, 53 Kawara-cho Shogoin, Sakyo-ku, Kyoto 606-8507, Japan

## Abstract

**Purpose:**

The purpose of this study was to investigate the feasibility of poly lactic/glycolic acid (PLGA) as a drug delivery carrier of Rho kinase (ROCK) inhibitor for the treatment of corneal endothelial disease.

**Method:**

ROCK inhibitor Y-27632 and PLGA were dissolved in water with or without gelatin (W1), and a double emulsion [(W1/O)/W2] was formed with dichloromethane (O) and polyvinyl alcohol (W2). Drug release curve was obtained by evaluating the released Y-27632 by using high performance liquid chromatography. PLGA was injected into the anterior chamber or subconjunctiva in rabbit eyes, and ocular complication was evaluated by slitlamp microscope and histological analysis.

**Results:**

Y-27632 incorporated PLGA microspheres with different molecular weights, and different composition ratios of lactic acid and glycolic acid were fabricated. A high molecular weight and low content of glycolic acid produced a slower and longer release. The Y-27632 released from PLGA microspheres significantly promoted the cell proliferation of cultured corneal endothelial cells. The injection of PLGA did not induce any evident eye complication.

**Conclusions:**

ROCK inhibitor-incorporated PLGA microspheres were fabricated, and the microspheres achieved the sustained release of ROCK inhibitor over 7–10 days in vitro. Our data should encourage researchers to use PLGA microspheres for treating corneal endothelial diseases.

## 1. Introduction

The corneal endothelium maintains corneal transparency by pump and leaky barrier functions [1]. The corneal endothelium is located at the anterior chamber side of the posterior cornea at a cell density of 2000–3000 cells/mm^2^ in a healthy subject. A phenotypical feature of corneal endothelial cells (CECs) is a severely limited proliferative ability [2]. When the corneal endothelium is impaired by any pathological conditions, trauma, or aging, the residual remaining CECs migrate and spread out to cover the impaired area, resulting in a cell density drop and increased polymorphism. The remaining CECs compensate the function to maintain corneal transparency, but once cell density drops to critical level, which is usually less than 500–1000 mm^2^/cells, the cornea loses its transparency [1].

Corneal transplantation using a donor cornea is the only therapeutic choice for treating corneal endothelial decompensation [3]. Researchers, including our group, have searched pharmaceutical agents such as EGF [4], PDGF [5], FGF-2 [6], lithium [7], and Rho kinase (ROCK) inhibitor [8], which can promote the proliferation of CECs, for the treatment of corneal endothelial diseases. Indeed, we performed pilot clinical research and showed that ROCK inhibitor eye drops have a potency for treating early stages of Fuchs endothelial corneal dystrophy and severe corneal endothelial damage induced by cataract surgery by enhancing the proliferation of residual CECs [9–11].

Poly lactic/glycolic acid (PLGA), a copolymer of poly lactic acid (PLA) and poly glycolic acid (PGA), has been intensively researched as a drug delivery carrier [12, 13]. During the degradation of PLGA for drug release, lactic acid and glycolic acid are coreleased and are both biologically removed by the surrounding cells through normal metabolic pathways [14]. The biocompatibility, low toxicity, and drug encapsulation capabilities of PLGA-based drug delivery systems have attracted the attention of researchers [12, 13]. Indeed, several therapies using PLGA have recently entered preclinical development [13, 15].

In the current study, we designed the PLGA microspheres, which incorporate ROCK inhibitor Y-27632, to enable efficient drug delivery for targeting corneal endothelium. We also evaluated the safety and stability of ROCK inhibitor released from PLGA microspheres through in vitro experiments. In addition, the safety of PLGA microsphere injection into the anterior chamber or subconjunctiva was evaluated in a rabbit model.

## 2. Materials and Methods

### 2.1. Microsphere Preparation

A selective ROCK inhibitor, Y-27632 (1 mg, Wako Pure Chemical Industries Ltd., Osaka, Japan), was dissolved in double-distilled water (200 *μ*L) with or without gelatin (20 *μ*g, Nitta Gelatin, Osaka, Japan) (W1) and poured into dichloromethane (2 mL, DCM, Nacalai Tesque, Kyoto, Japan) in which PLGA or poly (L-lactic acid) (PLA) (90 mg) was dissolved (O). The first inner W1/O emulsion was prepared by agitating for 60 secs using a vortex mixer at room temperature, followed by sonication for 300 secs using a UD-21P ultrasonic generator (Tomy Seiko Co. Ltd., Tokyo, Japan). The first emulsion was then poured into 1% polyvinyl alcohol (2 mL, PVA, Japan Vam & Poval Co. Ltd., Osaka, Japan) solution (W2), followed by vigorous mixing using a vortex mixer for 60 sec. This procedure permitted the formation of the double emulsion [(W1/O)/W2], in which the W1 phase was homogeneously dispersed in the O phase. The resulting double emulsion was poured into 1 wt% PVA solution (300 mL) and continuously stirred for 3 hours at 4°C until DCM was completely evaporated. The microspheres were washed several times with double-distilled water by centrifugation and freeze-dried into powdered microspheres. The microspheres with different composition ratios of lactic acid/glycolic acid were prepared similarly (Table 1).

The shape and size of the microspheres was evaluated using light microscopy (CKX41, Olympus Corp., Tokyo, Japan) and a micro scale (OB1, 1 mm scale length, 100 divisions, 0.01 mm pitch, MeCan Imaging Inc., Saitama, Japan). For scanning electron microscopy analysis, microspheres were fixed on an aluminum support with carbon-adhesive glue and coated with a 10 nm thick coating of gold-palladium (30 mA, 40 sec) (JSM 6701F; JEOL, Tokyo, Japan). The samples were observed using a scanning electron microscope (S-2380N, Hitachi Ltd., Tokyo, Japan).

### 2.2. Release Study

The Y-27632 release profiles from the PLGA microspheres were determined in vitro. PLGA microspheres in which Y-27632 (10 mg) was incorporated were incubated in a tube containing 1.0 mL phosphate-buffered saline solution (PBS; Nissui Pharmaceutical Co. Ltd., Tokyo, Japan) (pH: 7.4) at 37°C in a heat block (ALB-221, Scinics Co., Tokyo, Japan). PBS including released Y-27632 was evaluated at 1, 3, and 6 hour(s) and 1 to 30 day(s) after incubation. The concentration of Y-27632 in the samples was determined by high performance liquid chromatography (HPLC; Prominence, Shimazu Corp., Kyoto, Japan) equipped with one pump, an auto sampler, and an ultra violet (UV) detector. Calibration curves were prepared for Y-27632 based on the UV absorbance peak area at 269 nm. These calibration curves were used for the estimation of in vitro drug release. The Y-27632 release profile was calculated as follows: (cumulative amount of Y‐27632 released)/(total incorporated Y‐27632) × 100.

### 2.3. Cell Cultures

Eight corneas from four cynomolgus monkeys (3–5 years of age; estimated equivalent human age, 5–20 years) housed at Nissei Bilis Co. Ltd. (Otsu, Japan) were used for the study. The corneas were harvested at the time of the euthanization of the monkeys for other research purposes. CECs were cultured according to a modified protocol reported previously [16]. Briefly, Descemet's membrane including CECs was stripped from the cornea and incubated with 0.6 U/mL of Dispase II (Roche Applied Science, Penzberg, Germany) for 60 minutes at 37°C. CECs were then seeded onto a culture plate (CECs from one cornea to one well of a 12-well culture plate) with a culture medium composed of Dulbecco's modified Eagle's medium (DMEM, Life Technologies Corp., Carlsbad, CA) supplemented with 10% fetal bovine serum 50 U/mL penicillin, 50 *μ*g/mL streptomycin, and 2 ng/mL basic fibroblast growth factor (bFGF; Life Technologies Corp.). ROCK inhibitor was not added to the culture medium when preparing CECs for experiments. When the cells reached confluence in 10 to 14 days, they were trypsinized with 0.05% trypsin-EDTA (Life Technologies Corp.) for 5 minutes at 37°C and passaged at ratios of 1 : 2 to 4. CECs obtained after the 4–7th passage that still maintained a hexagonal and monolayer sheet-like structure were used for the experiments.

### 2.4. In Vitro Assessment of Y-27632 Released from Microspheres

To evaluate the stability and safety of Y-27632 released from the PLGA/PLA microspheres, the effect of released Y-27632 on cell growth of CECs was evaluated. Y-27632 incorporating PLGA microspheres (0020) were incubated in PBS, and PBS was recovered at 3 and 7 days after incubation. The concentration of Y-27632 was then evaluated by HPLC, and the recovered PBS including the released Y-27632 was added to the culture medium at a final concentration of Y-27632 of 10 *μ*M. Cultured monkey CECs were seeded at a density of 5.0 × 10^3^ cells/cm^2^ per well on a 96-well plate for 24 hours and then subjected to serum starvation for an additional 24 hours in the presence or absence of fresh Y-27632 (10 *μ*M) or Y-27632 released from PLGA microspheres after 3 or 7 days (10 *μ*M). The number of viable cells was determined by use of the CellTiter-Glo® Luminescent Cell Viability Assay (Promega, Fitchburg, Wisconsin) performed in accordance with the manufacturer's protocol. The number of CECs at 24 hours after treatment with Y-27632 was measured using the Veritas™ Microplate Luminometer (Promega, Fitchburg, Wisconsin).

### 2.5. Safety Assessment of Microspheres in Rabbit

To evaluate the safety of PLGA/PLA microspheres, 1 or 10 mg PLGA microspheres (0020) suspended in 200 *μ*L PBS were injected into the anterior chamber and 1 mg PLGA microspheres suspended in 200 *μ*L PBS were injected into the subconjunctiva of the 9 right eyes of 9 rabbits (*n* = 3). The left eyes were used as a control. The corneal appearance of the 9 rabbits was examined at 1, 2, 3, 5, 7, 10, and 14 days using a slitlamp microscope, and corneal opacification and conjunctival hyperemia were evaluated according to the grading system (Supplemental Table 1 available online at https://doi.org/10.1155/2017/1598218) modified from the previous report [17]. Intraocular pressure was determined with a Tonovet^®^ (Icare Finland, Vantaa, Finland) instrument. Corneal thickness was determined through the use of an ultrasound pachymeter (SP-2000; Tomey, Nagoya, Japan).

### 2.6. Histological Examination of Rabbit Eyes after Poly Lactic/Glycolic Acid (PLGA) Microsphere Injection

After 14 days of PLGA microsphere injection, rabbits were euthanized, the eye balls were enucleated, and sclerocorneal specimens were prepared using ophthalmic surgical scissors. Samples were fixed in 4% formaldehyde and incubated for 30 minutes in 1% bovine serum albumin to block nonspecific binding. The samples were then incubated overnight at 4°C with antibodies against Na^+^/K^+^-ATPase (1 : 300, Upstate Biotechnology, Lake Placid, NY), ZO-1 (1 : 300, Life Technologies Corp.), and connexin 43 (1 : 300, Life Technologies Corp.). Alexa Fluor® 488-conjugated goat anti-mouse (Life Technologies Corp.) was used as a secondary antibody at a 1 : 1000 dilution. Cell morphology was evaluated after actin staining with a 1 : 400 dilution of Alexa Fluor 594-conjugated phalloidin (Life Technologies Corp.). Nuclei were stained with 4′,6-diamidino-2-phenylindole (DAPI, Dojindo Laboratories, Kumamoto, Japan). The samples were examined with a fluorescence microscope (TCS SP2 AOBS; Leica Microsystems, Wetzlar, Germany).

### 2.7. Statistical Analysis

The statistical significance (*p* value) of differences in the mean values of the two-sample comparison was determined with the Student's *t*-test. The statistical significance of comparisons of multiple sample sets was analyzed with Dunnett's multiple-comparisons test. *p* < 0.05 was considered as statistically significant. Results were expressed as mean ± standard error of the mean.

## 3. Results

### 3.1. Characteristics of Poly Lactic/Glycolic Acid (PLGA) or Poly Lactic Acid (PLA) Microspheres for Y-27632 Release

**Figure 1:**
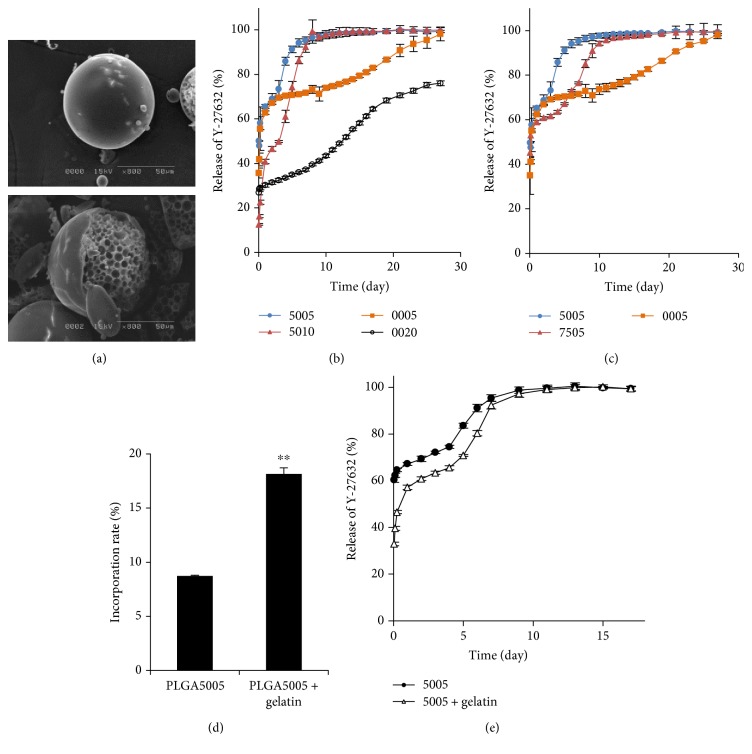
Characteristics of poly lactic/glycolic acid (PLGA) or poly lactic acid (PLA) microspheres for Y-27632 release. (a) PLGA microspheres were examined with a scanning electron microscope. The upper panel shows the surface of the microsphere, and the lower panel shows the internal structure of the microsphere. (b) PLGA or PLA microspheres were incubated in a PBS at 37°C for 14 days, and the amount of Y-27632 released was determined by HPLC. The effect of the molecular weight on the release profile for PLGA or PLA microspheres was evaluated. The cumulative amount of released Y-27632 was plotted in the graph (*n* = 3). (c) PLGA or PLA microspheres were incubated in PBS at 37°C, and the amount of Y-27632 released was determined by HPLC. The effect of the composition of lactic acid and glycolic acid on the release profile was evaluated (*n* = 3). The PLGA5005 release curve plotted is the same as that shown in Figure 1(b). (d) Y-27632 was dissolved in ultrapure water with or without gelatin, and PLGA5005 microspheres were prepared (*n* = 3). The incorporation rate was evaluated by HPLC. ^∗∗^*p* < 0.01. (e) PLGA5005 with or without gelatin that incorporated Y-27632 was incubated at PBS at 37°C for 14 days, and the amount of Y-27632 released was determined by HPLC. Three independent experiments were performed.

Y-27632-incorporated PLGA or PLA microspheres with different molecular weights and different composition ratios of lactic acid and glycolic acid were prepared (Table 1). All PLGA or PLA microspheres exhibited similar morphology and size. SEM showed that representative images of PLGA microspheres were spherical with a smooth surface (Figure 1(a), upper panel). The inside of the microspheres was porous core due to double emulsion, which is designed to incorporate Y-27632 (Figure 1(a), lower panel). We first evaluated the effect of molecular weight on the release profile in PLGA or PLA microspheres. PLGA5005 had released 65.5 ± 0.9% (approximately 8.22 *μ*g) of the Y-27632 by 1 day and 98.7 ± 0.6% (approximately 12.4 *μ*g) by 14 days, while PLGA5010, which has a higher molecular size than PLGA5005, released 40.8 ± 1.0% (approximately 0.56 *μ*g) of Y-27632 by 1 day and 99.1 ± 1.7% (approximately 1.37 *μ*g) by 14 days (*p* < 0.01 at 1 day). PLA0005 released 62.8 ± 0.8% (approximately 5.86 *μ*g) of Y-27632 by 1 day and 77.7 ± 0.5% (approximately 7.25 *μ*g) by 14 days, while PLA0020, which has a higher molecular size than PLA0005, released 30.2 ± 0.9% (approximately 0.64 *μ*g) of Y-27632 by 1 day and 55.3 ± 0.6% (approximately 1.16 *μ*g) by 14 days (*p* < 0.01 at both 1 day and 14 days) (Figure 1(b)). These data showed that a lower molecular weight increased the rate of drug release at a fixed composition ratio of lactic acid and glycolic acid. We next evaluated the effect of the composition of lactic acid and glycolic acid on the release profile. PLGA7505 released 58.9% of Y-27632 at 1 day and 97.4% at 14 days, and PLA0005 released 62.4% of Y-27632 at 1 day and 77.5% at 14 days. This data showed that a smaller amount of glycolic acid made the release slower and a smaller amount of lactic acid made the release of Y-27632 faster (Figure 1(c)).

The mean incorporation rate of Y-27632 was 8.74% in PLGA microspheres, though PLGA microspheres prepared with gelatin enabled a significantly higher incorporation of Y-27632 at 18.1%, showing that gelatin reduced the loss of ROCK inhibitor during preparation of the PLGA microspheres (Figure 1(d)). Characteristic release curves showed that both PLGA with or without gelatin have the potency to release Y-27632 and that the initial burst release of Y-27632 tended to decrease for microspheres with gelatin (Figure 1(e)).

### 3.2. In Vitro Evaluation of Y-27632 Released from Microspheres

**Figure 2:**
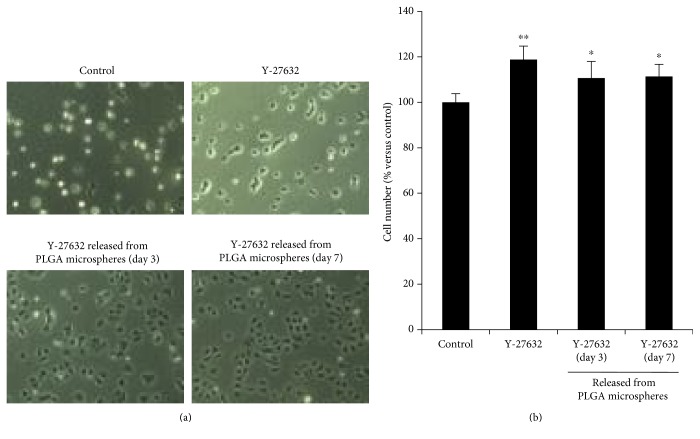
Stability and safety assessment of Y-27632 released from PLGA microspheres. (a) Y-27632 incorporating poly lactic/glycolic acid (PLGA) microspheres was incubated in PBS, and PBS was recovered after 3 and 7 days. The amount of Y-27632 released in the PBS was added to a culture medium as final concentration of Y-27632 as 10 *μ*M. Phase contrast images of corneal endothelial cells (CECs) cultured for 24 hours are shown. (b) CECs were seeded at a density of 5.0 × 10^3^ cells/cm^2^ per well on a 96-well plate for 24 hours and subjected to serum starvation for an additional 24 hours supplemented with fresh Y-27632 (10 *μ*M) or Y-27632 released from PLGA microspheres after 3 or 7 days (10 *μ*M). Control cells were CECs cultured for an additional 24 hours without supplementation with Y-27632. The data represent average ± SE (*n* = 5). ^∗∗^*p* < 0.01, ^∗^*p* < 0.05. These experiments were performed in triplicate.

The effect of Y-27632 released from PLGA microspheres on CEC proliferation was evaluated. Phase contrast images showed that CECs cultured with a culture medium supplemented with fresh Y-27632 (10 *μ*M) showed higher numbers of cells than the control. The Y-27632 released from the PLGA microspheres was recovered after incubation and was added to a growth medium at a final concentration of 10 *μ*M. Higher numbers of CECs were observed than those in the control after 24 hours of cultivation with those growth media that were supplemented with Y-27632 released from PLGA microspheres (Figure 2(a)). The cell numbers of CECs were significantly increased by the supplementation of fresh Y-27632 or the Y-27632 recovered from PLGA microspheres after an incubation of 3 and 7 days (118.8%, 110.6%, and 111.4%, resp.) (Figure 2(b)). These results showed that Y-27632 incorporated in PLGA microspheres was released and its biological features were maintained.

### 3.3. In Vivo Safety Evaluation of Microspheres

**Figure 3:**
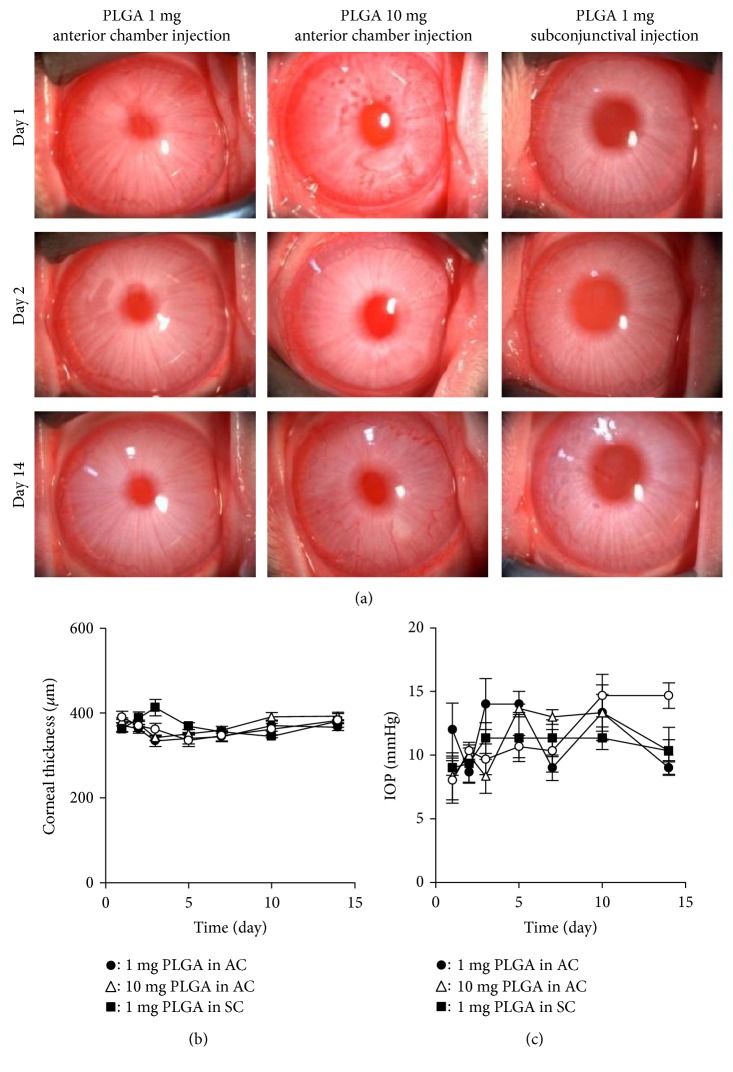
Safety assessment of poly lactic/glycolic acid (PLGA) microspheres in a rabbit eye. (a) One or 10 mg PLGA microspheres suspended in 200 *μ*L phosphate buffer solution (PBS) was injected into the anterior chamber, and 1 mg PLGA microspheres suspended in 200 *μ*L PBS was injected into the subconjunctiva. Nine right eyes of 9 rabbits were used for the experiments (*n* = 3). Anterior segments were evaluated by slitlamp microscopy for 14 days. (b, c) Corneal thickness and intraocular pressure were evaluated for 14 days and are shown in the graph. The central corneal thickness was evaluated by ultrasound pachymetry. Intraocular pressure was determined with a Tonovet.

We evaluated the feasibility of using PLGA microspheres in the eye by conducting a study using healthy rabbit eyes. Slitlamp microscopy showed that no eye complications, such as conjunctival injection, corneal opacity, cataract formation, or severe inflammation, occurred in any of the groups (Figure 3(a)). Corneal opacification and conjunctival hyperemia scores were zero in all of the eyes for all of the groups in which PLGA microspheres were injected into the anterior chamber (1 mg or 10 mg) and into the subconjunctiva (1 mg) (data not shown). Some PLGA microspheres were visible by slitlamp microscopy examination, especially in the iris, but no aggregation of microspheres was observed in the anterior chamber. Corneal thickness and intraocular pressure were not altered to abnormal levels during the 14-day observation period (Figures 3(b) and 3(c)).

**Figure 4:**
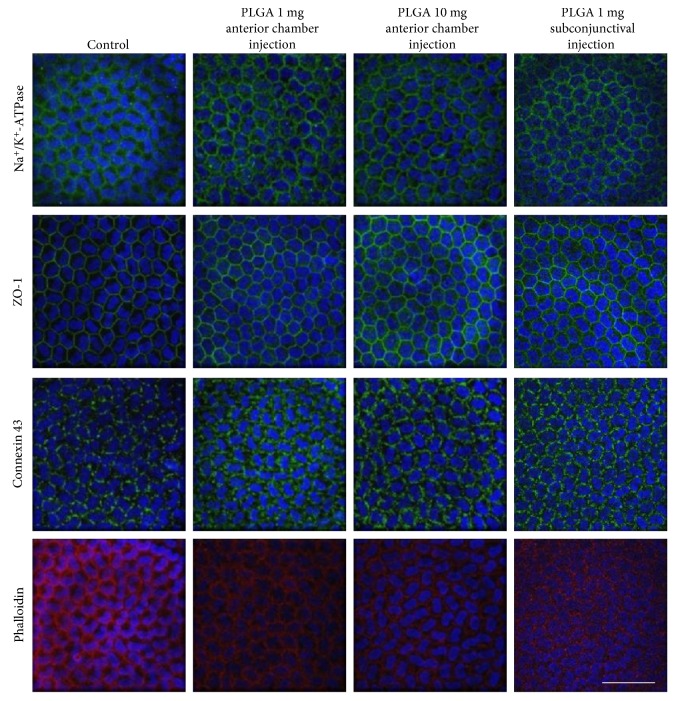
Histological evaluation of corneal endothelium in rabbit eyes injected with poly lactic/glycolic acid (PLGA) microspheres. One or 10 mg PLGA microspheres was injected into the anterior chamber, and 1 mg PLGA microspheres was injected into the subconjunctiva. After 14 days, corneal endothelium was evaluated by immunofluorescence staining and phalloidin staining. Na^+^/K^+^-ATPase (a marker of pump function), ZO-1 (a marker of tight junction), and connexin 43 (a marker of gap junctions) were used to evaluate the functional property of corneal endothelium. Phalloidin staining was used for morphological analysis. Nuclei were stained with DAPI. Scale bar: 50 *μ*m.

Corneal endothelium of eyes in which PLGA microspheres were injected into the anterior chamber and the subconjunctiva expressed Na^+^/K^+^-ATPase (a marker of pump function), ZO-1 (a marker of tight junction), and connexin 43 (a marker of gap junctions) as did the control. Phalloidin staining showed that the cell morphology was hexagonal and monolayer in PLGA microsphere-treated eyes as with the control, and no morphological change was observed (Figure 4).

## 4. Discussion

Bullous keratopathy caused by cataract surgery is one of the leading causes of corneal transplantation due to corneal endothelial decompensation [3]. The Eye Bank Association of America reported that of the 28,394 corneal transplantations performed in 2015, 15,707 (55.3%) were for corneal endothelial decompensation [18]. In terms of bullous keratopathy due to severe corneal endothelial damage by cataract surgery, 8290 (29.2%) corneal transplantations were performed in the United States [18]. Likewise, 20–40% of corneal transplantations are performed to treat bullous keratopathy caused by cataract surgery in Asian countries [19–21].

In 2009, we reported that the ROCK inhibitor Y-27632 promoted cell proliferation of cultured CECs [8]. We subsequently demonstrated that the topical application of ROCK inhibitor such as Y-26732 and ripasudil as a form of eye drops enhances wound healing of the cornea endothelium by promoting cell proliferation of CECs in rabbit and monkey models [10, 11, 22, 23]. Based on these findings, we performed pilot clinical research in which patients with corneal decompensation were treated with Y-27632 eye drops following to a transcorneal freezing procedure to partially remove the damaged cornea endothelium. Y-27632 eye drops showed effectiveness in reducing the central corneal thickness in patients with early stage Fuchs endothelial corneal dystrophy [9, 10]. In addition, we demonstrated that ROCK inhibitor eye drops can enhance the wound healing of damaged corneal endothelium caused by cataract surgery, thus successfully avoiding the need for corneal transplantation [11]. Although further randomized clinical trials are necessary, ROCK inhibitor currently might be one of the most promising pharmaceutical agents that can be applied for corneal decompensation.

Possible complications of the injection of PLGA into the anterior chamber are direct mechanical damage to the corneal endothelium or lens, indirect damage to the corneal endothelium by biodegradation products from PLGA, and intraocular pressure elevation by inhibiting aqueous humor outflow. We therefore conducted a safety assessment using healthy rabbit eyes to evaluate the feasibility of applying PLGA microspheres for intracameral injection. Here, we showed that the injection of PLGA into the anterior chamber or the subconjunctiva did not induce any evident complications. No inflammatory response was observed, in agreement with the reported biocompatibility safety profile of PLGA. PLGA is capable of encapsulating a wide range of drugs of almost any molecular size [12]. Our in vitro study consistently showed that ROCK inhibitor released from PLGA promoted the proliferation of CECs in the same manner as unencapsulated ROCK inhibitor, suggesting that ROCK inhibitor was incorporated into and released from PLGA without damage to the molecular properties of the inhibitor. Further study is needed, but it is suggested that PLGA might be applicable as a drug carrier that is injected into the anterior chamber.

In our clinical research, we suggested that a ROCK inhibitor has the potency to promote the cell proliferation of residual relatively healthy CECs [9–11] and that there is a “golden time” for a ROCK inhibitor, when the wounded space has not yet been covered by the migration and spreading of residual CECs associated with a cell density drop [24]. We showed that ROCK inhibitor eye drops restored corneal clarity within one week in patients with Fuchs endothelial corneal dystrophy following a transcorneal freezing procedure and within 1-2 months in patients with severe corneal endothelial damage due to cataract surgery. Therefore, in this study, we aimed to fabricate a PLGA system that would release Y-27632 for 7 to 10 days to improve the therapeutic efficacy during this window of opportunity; however, the release time should be optimized by further studies. The drug release from PLGA with degradation is influenced by various factors such as the initial molecular weight, the monomer composition rate of PLA and PGA, the drug type, and the pH of the surrounding circumstances [12, 25]. Among these factors, the molecular weight and the composition rate of PLA and PGA are recognized as important and are also easily controlled during fabrication [12]. Here, we showed that PLGA5010 exhibited a slower release than PLGA5005 and that PLA0020 exhibited slower release than PLA 0005, showing that PLGA or PLA microspheres with a high molecular weight produce slower and more prolonged Y-27632 release. This may be because polymers with lower molecular weights are less hydrophobic, which increases the rate of water absorption, consequent hydrolysis, and erosion [26]. The composition of PLA and PGA in PLGA is also an important factor influencing polymer degradation. In this study, PLGA7505 showed a slower release of Y-27632 than PLGA5005, and PLA0005 showed the slowest and longest release, although it exhibited an initial burst in release. This suggests that a lower content of GA in PLGA results in a slower and longer-term drug release from the PLGA microspheres. It has been demonstrated that the PGA : PLA ratio of 50 : 50 make PLGA the most hydrophilic and amorphous when compared to other ratios, resulting in faster degradation and drug release [12].

Although the further optimization of the release profile of the ROCK inhibitor is needed before clinical application, our results offers fundamental information for modifying the molecular weights and composition rates of PLA and PGA. Various mathematical, computational, and theoretical models may be applicable for the optimization of the release profile [27–29]. However, drug release from microspheres differs in the in vitro versus in vivo conditions, in part due to immunological responses and the plasticizing effects of biological substances [12]. For instance, the release of thymosin alpha 1 from PLGA was slightly faster in an in vivo assay than in an in vitro assay [30]. Conversely, other researchers have shown that PLGA degradation was slower in vivo than in vitro [31]. Therefore, pharmacokinetics studies are needed on the release of ROCK inhibitor in the anterior chamber. The drawback of this study is the lack of in vivo experiments that show an enhancement of corneal endothelial wound healing by ROCK inhibitor incorporated into PLGA. The fate of the PLGA injected into the anterior chamber is also not well characterized, and further in vivo experiments are needed prior to initiating clinical applications. In the clinical setting, however, the treatment of corneal endothelial damage induced by cataract surgery would seem to be an important target for this PLGA therapy.

In conclusion, we fabricated ROCK inhibitor-incorporated PLGA microspheres, and those microspheres act as carrier for the sustained release of the ROCK inhibitor over 7–10 days. In addition, the PLGA microspheres did not exhibit any evident complication in the eyes of a rabbit model. Although further optimization of the release profile of PLGA and in vivo experiments for safety and effectiveness assessment are required, PLGA microspheres would appear to represent a promising drug delivery carrier for ROCK inhibitor.

## Supplementary Material

SUPPLEMENTAL TABLE 1. Grading system for corneal opacification and conjunctival hyperemia modified from Sotozono et al. [17].

## Figures and Tables

**Table 1:** Summary of poly lactic/glycolic acid (PLGA) or poly lactic acid (PLA) microspheres.

Type	Molecular weight	Composition ratio	Drug incorporation rate (%)	Particle size (*μ*m)
		Lactic acid : glycolic acid		
5005	5000	50 : 50	12.5	12.63 ± 5.76
5010	10000	50 : 50	1.38	11.83 ± 6.85
7505	5000	75 : 15	1.17	14.30 ± 5.13
0005	5000	100 : 0	9.33	16.44 ± 6.09
0020	20000	100 : 0	2.11	13.94 ± 7.32
5005 + gelatin	5000	50 : 50	18.1	11.80 ± 7.74
