# Supplementary material for: Development of Poly Lactic/Glycolic Acid (PLGA) Microspheres for Controlled Release of Rho-Associated Kinase Inhibitor
Source: J Ophthalmol. 2017 Jul 27;2017:1598218. doi: 10.1155/2017/1598218 (PMC5551544; doi:10.1155/2017/1598218)
Supplement: Supplementary file 1 — SUPPLEMENTAL TABLE 1. Grading system for corneal opacification and conjunctival hyperemia modified from Sotozono et al. [17]. [file 1598218.f1.pptx]

## Slide 1
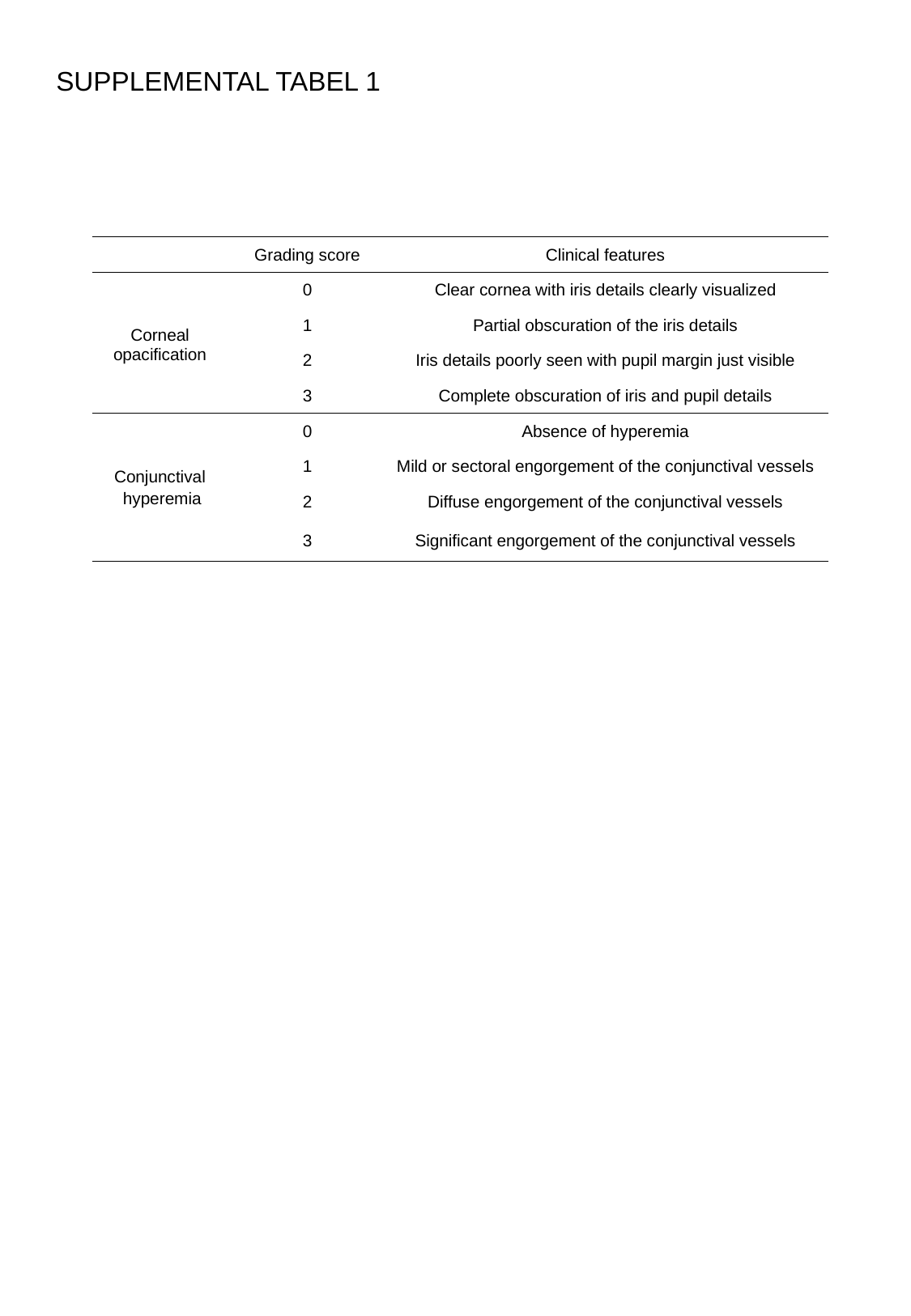

SUPPLEMENTAL TABEL 1
| | Grading score | Clinical features |
| --- | --- | --- |
| Corneal opacification | 0 | Clear cornea with iris details clearly visualized |
| | 1 | Partial obscuration of the iris details |
| | 2 | Iris details poorly seen with pupil margin just visible |
| | 3 | Complete obscuration of iris and pupil details |
| Conjunctival hyperemia | 0 | Absence of hyperemia |
| | 1 | Mild or sectoral engorgement of the conjunctival vessels |
| | 2 | Diffuse engorgement of the conjunctival vessels |
| | 3 | Significant engorgement of the conjunctival vessels |
